# Asociación entre SARS-COV-2 y enfermedades crónicas en personal de salud. Un programa de medicina preventiva

**DOI:** 10.15446/rsap.V25n2.105359

**Published:** 2023-03-01

**Authors:** Daniel Y. Chavarría-Castro, Estefanía Reyes-Varón, Angélica M. Salgado-Cordero, Irene Irisson-Mora, Luis E. Morales-Bartolo, Fabiola Álvarez-Montero

**Affiliations:** 1 DC: MD. Esp. Epidemiología. Unidad de Vigilancia Epidemiológica Hospitalaria, Hospital Juárez de México. Ciudad de México, México. dr.daniel-yair.c@hotmail.com Unidad de Vigilancia Epidemiológica Hospitalaria Hospital Juárez de México Ciudad de México México; 2 ER: MD. Medicina Preventiva. Instituto Nacional de Enfermedades Respiratorias (INER). Ciudad de México, México. fany-0116@hotmail.com Medicina Preventiva Instituto Nacional de Enfermedades Respiratorias (INER) Ciudad de México México; 3 AS: MD. Medicina Preventiva. Instituto Nacional de Enfermedades Respiratorias (INER). Ciudad de México, México. annsalgadoc@gmail.com Medicina Preventiva Instituto Nacional de Enfermedades Respiratorias (INER) Ciudad de México México; 4 II: MD. Esp. Endocrinología. Medicina Preventiva, Instituto Nacional de Enfermedades Respiratorias (INER). Ciudad de México, México. irene_irisson@hotmail.com Endocrinología. Medicina Preventiva Instituto Nacional de Enfermedades Respiratorias (INER) Ciudad de México México; 5 LM: MD. Medicina Preventiva, Instituto Nacional de Enfermedades Respiratorias (INER). Ciudad de México, México. Medicina Preventiva Instituto Nacional de Enfermedades Respiratorias (INER) Ciudad de México México; 6 FA: MD. Esp. Epidemiología. Unidad de Vigilancia Epidemiológica Hospitalaria, Hospital Regional No. 2, Instituto Mexicano del Seguro Social. Ciudad de México, México. resi_alva19@hotmail.com Instituto Mexicano del Seguro Social Epidemiología Unidad de Vigilancia Epidemiológica Hospitalaria Instituto Mexicano del Seguro Social. Ciudad de México Mexico

**Keywords:** COVID-19, SARS-CoV-2, obesidad, sobrepeso, medicina preventiva, personal de salud, vacunación *(fuente: DeCS, BIREME)*, COVID-19, SARS-CoV-2, obesity, overweight, preventive medicine, health personnel, vaccination *(source: MeSH, NLM)*

## Abstract

**Objetivo:**

La infección por SARS-CoV-2 ha sido relacionada con hipertensión, obesidad y diabetes para riesgo de hospitalización y muerte. Con respecto a las enfermedades pulmonares obstructivas, la literatura es diversa; hay variación en la frecuencia de estas y por ende en su relación con la COVID-19. La evidencia disponible únicamente detalla a los pacientes hospitalizados, y es escasa la referida a trabajadores de la salud, por lo que el analizar las principales comorbilidades en este grupo resulta de ayuda para la implementación de programas preventivos. El objetivo de este estudio fue describir la prevalencia y la asociación de diversas comorbilidades con la infección por SARS-CoV-2 en trabajadores de la salud.

**Métodos:**

Estudio transversal analítico, en trabajadores del principal centro de referencia nacional para enfermedades respiratorias de México, que acudieron a atención para descarte de infección por SARS-CoV-2 mediante un programa preventivo; para el tamaño de muestra se usó fórmula para cálculo de proporciones. Se analizaron medidas de resumen y asociación.

**Resultados:**

La prevalencia de COVID-19 fue de 22,9 %. Las comorbilidades más frecuentes en cuanto a prevalencia fueron: sobrepeso (29,0 %), obesidad (13,2 %), tabaquismo (8,6 %), hipertensión (5,5 %), rinitis alérgica (3,9 %) y asma (2,8 %). El sobrepeso, la obesidad y la vacunación contra SARS-CoV-2 tuvieron razones de momios para prevalencia de 1,78, 1,72 y 0,43, respectivamente. Las personas vacunadas y con comorbilidades tienen menor duración de la enfermedad (p=0,001).

**Conclusiones:**

La obesidad y el sobrepeso muestran asociación con SARS-CoV-2, la vacunación es un factor protector, sobre todo en aquellos pacientes con comorbilidades.

## Abstract (en)

**Objective:**

The SARS-CoV-2 infection has been linked to hypertension, obesity and diabetes as risks of hospitalization and death. Regarding obstructive pulmonary diseases, the scientific literature is diverse; finding variation in the frequency of these and therefore their relationship with COVID-19. The available evidence only details hospitalized patients, with insufficient information referring to health workers, so analyzing the main comorbidities in this group is helpful for the implementation and improvement of preventive programs. The objective of this study was to describe the prevalence and association of comorbidities with SARS-CoV-2 infection in health care workers.

**Methods:**

Analytical cross-sectional study, in workers of the main national reference center for respiratory diseases in Mexico, who came to care to rule out SARS-CoV-2 infection through a preventive program; calculation of proportions was performed for the sample size. Summary and association measures were calculated.

**Results:**

The prevalence of COVID-19 was 22.9 %. The most frequent comorbidities were: overweight obesity, smoking, hypertension, allergic rhinitis and asthma, with prevalences of 29.0 %, 13.2 %, 8.6 %, 5.5 %, 3.9%, 2.8 % respectively. Overweight, obesity and vaccination against SARS-CoV-2 had Prevalence Ratios of 1.78, 1.72 and 0.43 respectively.

**Conclusions:**

Obesity and overweight show association with SARS-CoV-2; vaccination is a protective factor, especially in people with comorbidities.

La infección por SARS-CoV-2, que causa la COVID-19, puede ser transmitida de persona a persona por gotas respiratorias 1, y constituye una amenaza global; del 14 al 20 de febrero del 2022 se reportaron 12 millones de casos nuevos y 67 000 defunciones, con un total de 422 millones de casos confirmados y 5,8 millones de defunciones a nivel mundial 2. En México, hasta el 22 de febrero del 2022, hubo 5 436 566 casos totales y 316 492 defunciones totales 3. Con el crecimiento de la pandemia, el acceso a equipo de protección personal en los trabajadores de la salud es una preocupación, debido a que corren el mayor riesgo de infección 4, lo que revela la importancia de programas de apoyo a los trabajadores por parte de las instituciones 5.

Del riesgo de hospitalización y muerte por COVID-19, en los Estados Unidos la hipertensión arterial fue la comorbilidad más frecuente, seguida de obesidad y diabetes 6,7; en México, la obesidad interviene en el efecto de la diabetes sobre la letalidad del COVID-19 8. La obesidad tiene relación como una de las comorbilidades más importantes asociadas a COVID-19, no obstante, no se han investigado todos los espectros de esta asociación 9. En relación con las enfermedades pulmonares obstructivas, la literatura es diversa; se ha documentado variación en la frecuencia entre países y por ende su relación con la COVID-19. En España, los pacientes con asma o EPOC no parecen expuestos a mayor riesgo 10. En China, una serie de 140 pacientes no describió casos de asma 11. En Italia, la frecuencia de EPOC fue del 4 % 12. Hay trabajos que describen dudas en la asociación del asma/EPOC, así como un mayor riesgo de hospitalización por COVID-19 al poseer alguna de estas manifestaciones 13. Otros países describen una frecuencia más elevada de dichas enfermedades 14. El tabaquismo se asoció a mayor gravedad para COVID-19 15,16. La evidencia disponible únicamente detalla los pacientes hospitalizados, siendo poca la referida a trabajadores de la salud, por lo que el analizar las principales comorbilidades en este grupo es de ayuda para la implementación y la mejora de programas preventivos. El objetivo fue describir la prevalencia y la asociación de las comorbilidades y la infección por SARS-CoV-2 en trabajadores de la salud.

## MÉTODOS

Se trato de un estudio transversal analítico, cuya población de estudio fueron los trabajadores del principal centro de referencia nacional para enfermedades respiratorias de México: el Instituto Nacional de Enfermedades Respiratorias/Ciudad de México (INER), que solicitaron atención para descarte de infección por SARS-CoV-2, mediante un programa específico, el cual brinda consulta médica a trabajadores, con fines preventivos y limitación de enfermedad y brotes en aquellos que acuden por síntomas o por ser contactos de casos positivos o sospechosos.

Se calculó el tamaño de la muestra, tomando en cuenta los años pandémicos por COVID-19 (del 2020 al 2022) y aplicando una fórmula para cálculo de proporciones mediante la herramienta *online* libre OpenEpi. Se fijó una frecuencia anticipada del 50 %, debido a la discrepancia en la frecuencia de la enfermedad en diversas fuentes, y se estableció un nivel de confianza del 95 % y una precisión del 3 %, lo que arrojó un cálculo de 837 individuos. Adicionalmente, se sumó un 20 % en caso de muerte estadística, por lo que el tamaño de muestra final fue de 1 004 individuos. Además, se realizó muestreo aleatorio simple, y el período estudiado fue del 16 de abril del 2020 al 25 de enero del 2022.

Se construyó una base de datos (con información del expediente electrónico, así como producto de una entrevista telefónica que se llevó a cabo el mismo día de la atención clínica, o al siguiente día al tener el resultado del hisopado mediante técnica de PCR-RT para SARS-CoV-2). Con los mismos datos, se hizo la búsqueda y el registro de las variables y las comorbilidades, de las cuales se tomaron en cuenta algunas estudiadas en la literatura con relación al tema, como diabetes (DM), hipertensión (HAS), obesidad, sobrepeso, asma y enfermedad pulmonar obstructiva crónica (EPOC), así como otras halladas en los registros de atención clínica en la atención al trabajador. Se hizo limpieza de la base de datos, posteriormente se realizó un análisis exploratorio general de las variables de estudio, los datos faltantes y los incorrectos.

Para las variables estudiadas se usaron porcentajes (el porcentaje de positividad se calculó de acuerdo con la técnica utilizada, avalada por la Secretaría de Salud de México 17, rangos mínimo-máximo e intercuartilar (RIQ), tasas y medidas de tendencia central, y se determinó la asociación utilizando pruebas de Chi cuadrada (Chi^2^) o exacta de Fisher, según correspondiera. Se usó una confianza al 95 % y se calculó el valor de *p.* En el caso de las variables que arrojaron un valor de *p* estadísticamente significativo (<0,05), se calculó la razón de momios para la prevalencia (RMP) y los intervalos de confianza al 95 % (IC95 %). Se compararon las diferencias en los días hasta la negativización entre las personas con y sin obesidad y sobrepeso y comorbilidades (para considerar como sobrepeso u obesidad se usaron los cortes recomendados por la Organización Mundial de la Salud), mediante prueba de Kruskal-Wallis (previa determinación de normalidad), y para analizar las diferencias entre los grupos con comorbilidades se usó la U de Mann-Whitney como prueba *post-hoc* (para dar de alta a un paciente y retornar a sus actividades, el programa otorga atención individualizada, dependiendo de los síntomas; se dan citas cada 7 a 14 días para un hisopado de control mediante RT-PCR). Adicionalmente, se analizaron los efectos de la vacunación contra COVID-19. El análisis estadístico se llevó a cabo con el software Stata 14. Este protocolo fue avalado por los respectivos comités de ética e investigación de la institución.

## RESULTADOS

La muestra quedó conformada por 953 trabajadores que solicitaron atención, de los que fueron descartados 51 por muerte estadística. Entre los sujetos estudiados, a 16 no se les tomó muestra para PCR, debido a que no contaban con suficientes factores para considerarse como sospechosos, no obstante, fueron incluidos porque se completó el protocolo de atención clínica para descarte de COVID-19. Los rangos mínimo y máximo de edad fueron 17 y 82 años (mediana de 32); el 36,7 % de los individuos estudiados fueron hombres y el 63,3 % mujeres. De los individuos de ambos sexos se obtuvieron 218 trabajadores positivos para SARS-CoV-2 (prevalencia de la enfermedad del 22,9 %, porcentaje de positividad general de 23,3 %). Se obtuvo un registro total de 497 individuos con comorbilidades y, así mismo, se obtuvieron 218 casos positivos para SARS-COV-2 y 546 individuos vacunados contra dicha enfermedad.

De acuerdo con la solicitud de atención clínica, enfermería fue la que representó la categoría con mayor porcentaje de atenciones (43,3 %), seguida del personal administrativo (14,3 %) y médico (18,1 %). Al analizar la prevalencia por categoría, se conservó el orden previamente mencionado, donde enfermería tiene la mayor frecuencia (9,6 %), seguida de administrativos (4,1 %) y médicos (3,7 %). En contraparte, se calculó la tasa de casos positivos de acuerdo al personal por categoría, y se observó una mayor relación en el personal de transporte (100 %), seguido del personal de lavandería (44,4 por cada 100 trabajadores) (Tabla 1).

**Tabla 1:** Tasas, porcentajes y prevalencia

Personal	Positivos	Población	%	Prevalencia	Tasa de positivos por categoría
Enfermería	92	413	43,3	9,6	22,3
Administrativos	39	136	14,3	4,1	28,7
Médicos	35	173	18,1	3,7	20,2
Inhaloterapeutas	10	32	3,4	1,0	31,2
Intendencia	9	42	4,4	0,9	21,4
Técnicos	8	20	2,1	0,8	40,0
Alimentación	5	29	3,0	0,5	17, 2
Lavandería	4	9	0,9	0,4	44,4
Camilleros	3	12	1,3	0,3	25,0
Investigadores	3	24	2,5	0,3	12,5
No identificado	2	11	1,1	0,2	18,2
Químicos	2	9	0,9	0,2	22,2
Transportistas	2	2	0,2	0,2	100,0
Bioseguridad	2	6	0,6	0,2	33,3
Laboratoristas	1	14	1,5	0,1	7,1
Vigilancia	1	5	0,5	0,1	20,0
Estomatología	0	3	0,3	0,0	0,0
Estudiantes	0	6	0,6	0,0	0,0
Psicología	0	4	0,4	0,0	0,0
Físicos	0	1	0,1	0,0	0,0
Trabajo Social	0	2	0,2	0,0	0,0

Los registros de pruebas tomadas al personal indican un mínimo y un máximo de 0 y 16, respectivamente; el promedio fue de 0,3 y la moda de 0,1. Esta última fue una repetición encontrada en 243 individuos.

Las comorbilidades más frecuentes encontradas en la población fueron: sobrepeso obesidad, tabaquismo, hipertensión, rinitis alérgica y asma, con prevalencias del 29,0 %, 13,2 %, 8,6 %, 5,5 %, 3,9 % y 2,9 %, respectivamente, de las cuales el sobrepeso y la obesidad tuvieron RMP de 1,78 y 1,62 (Tabla 2).

**Tabla 2:** Relación entre COVID-19 y enfermedades crónicas

Enfermedad	Total	Prevalencia	*P*	RMP	IC 95 %
Cardiopatías^**^	12	1,2	1,00	---	---
HAS^*^	52	5,5	0,52	---	---
Dislipidemia^*^	8	0,8	0,39	---	---
DM^*^	31	3,2	0,96	---	---
Sobrepeso^*^	275	28,9	>0,001	1,78	1,27-2,48
Obesidad^*^	126	13,2	0,020	1,62	1,04-2,48
EPOC^*^	0	-	---	---	---
Tabaquismo^*^	82	8,6	0,37	---	---
Rinitis^*^	37	3.9	0,54	---	---
Asma^*^	27	2,8	0,76	---	---
Insuficiencia venosa^**^	6	0,6	0,62	---	---
Hipotiroidismo^*^	25	2,6	0,89	---	---
Embarazo^**^	3	0,3	0,54	---	---
Vacuna ^*^	75	7,9	>0,001	0,43	0,31-0,59

^*^Exacta de Fisher; ^*^Chi2.

**Figura 1:**
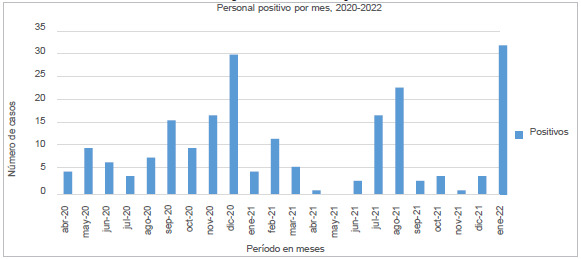
Gráfica de contagios

La gráfica de distribución en el tiempo muestra las "oleadas" de enfermedad entre el personal de salud, con cuatro grandes repuntes (Figura 1).

En cuanto a la relación de la vacuna contra SARS-CoV-2, de acuerdo a los estatutos nacionales en el lapso de estudio, durante el 2020 y el 2021 se inició la estrategia nacional de vacunación para el personal de salud. En dicho periodo se encontró una población vacunada de 546 individuos, lo que equivale a una tasa de vacunación del 57 % de los trabajadores, que a su vez equivale al personal clasificado como "primera línea" (personal en atención clínica directa con pacientes COVID-19) para al menos una dosis. Las vacunas aplicadas fueron en su mayoría Pfizer-BioNTech, con 474 dosis (86,8 %), seguida de AztraZeneca con 65 dosis (11,9 %), Sinovac y Cansino con tres dosis cada una (0,5 %) y por último Spuntik-V con una dosis (0,2 %).

Al analizar la asociación entre dichas variables en función de conocer si el haberse vacunado fungía como un factor protector, se encontró una asociación estadísticamente significativa p=>0,001, RMP=0,43, IC95 %= 0,31-0,59.

Al estudiar el tiempo de duración de la enfermedad (demostrada por PCR-RT), considerando el peso corporal, no hay diferencias estadísticamente significativas; no obstante, luego de confrontar a los que se vacunaron antes de padecer COVID-19 con aquellos que no lo hicieron, se encontró significancia estadística (p>0,001).

En el análisis *post-hoc,* los vacunados previamente a la enfermedad documentada por COVID-19 tuvieron una menor duración de la enfermedad, con una mediana de 14 días (RIQ de 9 días) en comparación con quienes no tenían antecedentes de vacunación antes de la infección; la prueba U de Mann-Whitney muestra diferencias estadísticamente significativas en los vacunados, así como en los vacunados, que además tienen una o más comorbilidades (Tabla 3).

**Tabla 3:** Tiempo de duración de la enfermedad en días

Estadístico	Sin comorbilidades	Con comorbilidades	Sin sobrepeso	Con sobrepeso	Sin obesidad	Con obesidad	Vacunados previamente	No vacunados previamente	Vacunados previamente y con comorbilidades	No vacunados previamente, con comorbilidades
Mediana	15	17	16	16	16	20.5	14	17	15	17.5
RIQ/ P25-75	8 (14-22)	14 (14-28)	11 (14-25)	14 (14-28)	11 (14-25)	15 (14-29)	9 (12,5-21,5)	14 (15-29)	9.5 (13-2,5)	17 (15-32)
p^*^	0,08		0,44		0,24		>0,001		0,001	

^*^ Análisis con U de Mann-Whitney.

## DISCUSIÓN

En nuestra población algunas enfermedades crónicas como la DM y la HAS tuvieron menor frecuencia a la reportada por fuentes oficiales 18, no obstante, se infiere que pudo existir subregistro, como es el caso de la EPOC, de la cual no se encontraron casos, aunque otros estudios refieren la falta de datos suficientes para abordar la relación EPOC-COVID-19. Las personas con sobrepeso tuvieron 1,78 veces el riesgo de infección por SARS-CoV-2, en comparación con quienes no tenían tal padecimiento. De la misma manera, las personas con obesidad tuvieron 1,62 veces el riesgo.

Al igual que en reportes europeos y asiáticos, en nuestra población los padecimientos respiratorios obstructivos no fueron tan frecuentes, aun cuando hay duda de su asociación con COVID-19, cuyo comportamiento es similar al de otras fuentes; en una serie de China de pacientes infectados por SARS-CoV-2, que tomó en cuenta enfermedades pulmonares, no se describieron casos de asma, en tanto que el 1,4 % de los pacientes tenía EPOC 11. En un estudio de Italia, Grasielli menciona una baja frecuencia de EPOC (del 4 %), y la cifra de asma fue tan baja que no se mencionó 12. En relación con el tabaquismo, nuestro estudio no demuestra una asociación con la enfermedad. Aunque no se encuentran tantos estudios que describan la relación entre los cigarrillos y la infección por SARS-CoV-2, hay trabajos que refieren asociación entre el tabaquismo activo y una mayor gravedad por COVID-19 15,16.

Las oleadas de casos concuerdan con lo descrito por la literatura de acuerdo a la distribución de la enfermedad, que corresponden a las semanas epidemiológicas de diciembre del 2020, febrero del 2021, julio del 2021 y diciembre del 2021 a enero del 2022 3. De la misma manera, la forma de la "curva" de casos positivos concuerda con lo hallado en otras fuentes oficiales que muestran una disminución de los casos debido a la variante Gamma, entre julio y agosto del 2020, para dar paso a un aumento en los casos de B 1.1.222 y B 1.1.519 19.

En este trabajo se encontró una RMP protectora en trabajadores (57 % menor riesgo de infección) con antecedente de vacunación contra SARS-CoV-2, lo que es concordante con la bibliografía que demuestra que hay una protección del 52 % después de al menos una dosis de la vacuna 20, lo que a su vez refuerza la importancia de la vacunación en la población que presenta una o más comorbilidades.

En cuanto al tiempo de negativización, concluimos que no hay diferencias entre las personas con y sin comorbilidades. No obstante, el haberse vacunado parece ser un factor para una menor duración de la positividad y de la enfermedad ante muestras consecutivas, lo que se ve reflejado en la mediana de la duración de positividad en días, la cual fue menor entre los grupos analizados con antecedente de vacunación. Por lo tanto, existen diferencias a favor de los trabajadores vacunados, lo que se observa al analizar a aquellos trabajadores que cuentan con una o más comorbilidades. En este trabajo, aunque los tiempos en días no son iguales, se concluye que los hallazgos son similares a otros autores, como Fei Zhou 21, que reporta una duración media de 20 días; una debilidad de este trabajo es que no se contó con datos suficientes para analizar y evaluar casos de inmunodepresión.

Los números de muestras para pruebas PCR-RT para SARS-CoV-2 hacen inferir que, en relación con la atención clínica, en este instituto se siguió un protocolo de atención basado en el trabajador de la salud, lo cual revela la importancia de los programas preventivos. Esto, aunado a todo lo anteriormente comentado y a que en esta población estudiada no hubo ocurrencia de defunciones por COVID-19 en los trabajadores, pone de manifiesto la importancia de contar con un programa de atención al trabajador que se base en la prevención y la mitigación de riesgos. Implementar este tipo de esquemas de trabajo en las instituciones de salud repercute en tener una baja frecuencia y tasas reducidas de enfermedad y mortalidad 22,23.

Como limitaciones de este estudio se debe señalar que hubo muerte estadística, correspondiente al 5% de los pacientes (los cuales fueron descartados), debido a que no se tenía información completa, no se hacía mención de los diagnósticos de interés, o no se habían interrogado. Para realizar el análisis de la vacuna como un posible factor protector se tomó en cuenta a aquellos que tenían un período mayor a 24 horas después de la aplicación de la primera dosis de vacuna. El tiempo de negativización en los positivos se analizó únicamente con 185 casos de trabajadores positivos, que eran los que tenían registros completos; en los restantes, no se encontraron los registros hasta la negativización en el respectivo expediente. Las muestras de hisopados PCR-RT no se genopitificaron, por lo cual, no fue posible conocer las variantes en tiempo y forma, puesto que el fin primario de este programa era la prevención y mitigación de brotes ♠
